# Simultaneous degradation of β‐cypermethrin and 3‐phenoxybenzoic acid by *Eurotium* *cristatum* ET1, a novel “golden flower fungus” strain isolated from Fu Brick Tea

**DOI:** 10.1002/mbo3.776

**Published:** 2018-12-12

**Authors:** Kaidi Hu, Weiqin Deng, Yuanting Zhu, Kai Yao, Jinyong Li, Aiping Liu, Xiaolin Ao, Likou Zou, Kang Zhou, Li He, Shujuan Chen, Yong Yang, Shuliang Liu

**Affiliations:** ^1^ College of Food Science Sichuan Agricultural University Ya’an Sichuan China; ^2^ College of Light Industry and Food Sichuan University Chengdu Sichuan China; ^3^ Institute of Food Processing and Safety Sichuan Agricultural University Ya’an Sichuan China; ^4^ College of Resources Sichuan Agricultural University Chengdu Sichuan China

**Keywords:** 3‐phenoxybenzoic acid, beta‐cypermethrin, biodegradation characteristics, *Eurotiumcristatum*, golden flower fungus

## Abstract

Beta‐cypermethrin (β‐CY) and its major metabolite 3‐phenoxybenzoic acid (3‐PBA) spread extensively in the environment because of utilization in agricultural and home formulations, exerting negative impact on environment as well as human health. Several golden flower fungi were isolated from fu brick tea, by which the biodegradation of β‐CY and 3‐PBA was evaluated, turning out strain *Eurotium cristatum* ET1 had the highest capacity. Furthermore, β‐CY and 3‐PBA degradation rates were positively correlated with biomass of *E*. *cristatum *ET1, and the processes of degradation fitted well with a first‐order kinetic equation. The half‐lives of β‐CY and 3‐PBA ranged from 3.382 to 11.517 days and 1.749 to 3.194 days, respectively, under different substrate concentrations, incubation temperatures, and pH values. The degraded products were analyzed using gas chromatography‐mass spectrometry and liquid chromatography‐mass spectrometry, and results showed that *E*. *cristatum *ET1 degrades β‐CY by transforming it into 3‐PBA, which is then gradually metabolized into phenol and catechol. Moreover, *E*. *cristatum* ET1 showed efficiency in degrading these metabolites. Our results suggest that this strain is a potential microorganism for bioremediation of pesticide‐contaminated environments and fermented foods.

## 1 INTRODUCTION

Agricultural practices have never been stopped from ancient times till now. As the pressure regarding population on agriculture is increasing, the pesticide usage also shows sustaining growing trend. (Liu, Pan, & Li, 2014; Stehle & Schulz, 2015; Yadav et al., 2015). Beta‐cypermethrin (β‐CY) is an important broad‐spectrum synthetic pyrethroid (SP) insecticide, which is widely used for insect control in public health, agricultural, and domestic applications since it is highly effective at low doses (Burr, 2014). Although β‐CY is considered safer than organophosphorous and carbamates, accumulating evidence has shown that β‐CY is harmful to the environment and human health due to its toxicity in several aspects (Soderlund et al., 2002; Wang et al., 2009). β‐CY is regarded as a possible human carcinogen by the Environmental Protection Agency of USA (Burr, 2014). With the increasing utilization of this insecticide, it leads to its bio‐accumulation through the food chain and consequently posing health risks to humans and nontarget organisms (Katagi, 2010). For instance, β‐CY has been frequently detected in serval kinds of agricultural product (especially in teas; Feng, Tang, Chen, & Li, 2015; Lozowicka et al., 2014; Parente et al., 2017). 3‐phenoxybenzoic acid (3‐PBA), a general degradation product of β‐CY and other SPs, exhibits higher mobility and stronger polarity than its parent compounds (Halden, Tepp, Halden, & Dwyer, 1999). Recent studies have suggested that 3‐PBA was not only persistent and refractory to natural degradation but also a major obstacle in β‐CY biodegradation because of its antimicrobial activities (Stratton & Corke, 1982). What is more, 3‐PBA is classified as an endocrine‐disrupting chemical and thus presents a greater risk than its parent compound (McCarthy, Thomson, Shaw, & Abell, 2006). It is also worth to mention that these two compounds have even been detected in human urine and elsewhere (Galea et al., 2015; Gosetti et al., 2018; Rousis, Zuccato, & Castiglioni, 2016). Hence, the removal of β‐CY and 3‐PBA from environment is urgent.

Biodegradation, widely applied in pesticide degradation (Fuchs, Boll, & Heider, 2011), is considered to be a low‐cost, effective, and safe alternative approach to degrade or eliminate β‐CY and 3‐PBA from environment. Several microorganisms have been reported harbor the capacity to simultaneously degrade β‐CY and 3‐PBA, during which some reactions in terms of hydrolyzation as well as oxidation were observed (Chen et al., 2014; Chen, Geng, Xiao, & Hu, 2012b; Chen, Hu, et al., 2011a; Tallur, Megadi, & Ninnekar, 2008). However, degradation of β‐CY and 3‐PBA by *Eurotium* spp. has not yet been reported, and most of these reported strains have been isolated from pesticide‐contaminated soils or sewage (Chen, Geng, et al., 2012b; Chen, Hu, et al., 2011a; Chen, Yang, Hu, & Liu, 2011b; Tallur et al., 2008), supposedly limiting their application in bioremediation in food processing.

Fu brick tea is one kind of traditional fully fermented black tea in China, which has been a necessary beverage for the ethnic groups living in the border regions of southern and western China throughout history, and its production and consumption are considerable (Xu et al., 2011). Although it has been reported that pesticide residues in fermentable tea are much lower than green tea (Feng et al., 2015; Regueiro, Lopez‐Fernandez, Rial‐Otero, Cancho‐Grande, & Simal‐Gandara, 2015), the reason for which is exactly unclear. In the present study, a novel golden flower fungus from fu brick tea was isolated, identified, and designated as *Eurotium cristatum* ET1 strain; this strain efficiently degraded both β‐CY and 3‐PBA. Biodegradation products of β‐CY were analyzed, and a possible biochemical degradation pathway was proposed. Our findings highlight the potential of *E*. *cristatum *ET1 for bioremediation of pesticide‐contaminated environment and fermented food.

## 2 MATERIALS AND METHODS

### 2.1 Chemicals and media

β‐cypermethrin (99.7%, purity), phenol (99%, purity), and catechol (99.9%, purity) standards were obtained from National Standard Material Center (Beijing, China). 3‐PBA (99%, purity) and 3‐phenoxybenzaldehyde (99%, purity) standards were purchased from Sigma‐Aldrich (Saint Louis, USA). Chromatographic grade acetonitrile was obtained from CNW Technologies GmbH (Dusseldorf, Germany). Technical grade β‐cypermethrin (97.2%, purity) was purchased from Nanjing Rongcheng Co., Ltd. (Nanjing, China). All other chemicals and solvents were of analytical grade. Stock solutions (5 mg/ml) of each standard or pesticide were prepared in ethanol.

Potato dextrose medium (PD) consisted of (per liter) 200 g of freshly peeled potatoes and 20 g of dextrose. Mineral salts medium (MM; pH 7.5) contained 1.5 g of (NH_4_)_2_SO_4_, 0.5 g of K_2_HPO_4_, 1.5 g of KH_2_PO_4_, 0.2 g of MgSO_4_, and 0.5 g of NaCl per liter water. Tween 80 (2 g/L) was added into both media.

### 2.2 Strain isolation and screening

Fu brick tea samples were collected from different tea factories located in Hunan province, China. Fungi were isolated by using a direct separation technique. Briefly, each brick was cut in half, and then gold particles on the surface were picked and inoculated on PDA (potato dextrose agar) plates. After incubation at 30°C, colonies of golden flower fungus that grew on plates were selected, purified, and maintained on PDA slants at 4°C.

Inocula were prepared by washing PDA slants with sterile spore diluent (composed of 0.2% Tween 80% and 0.1% agar) after these strains were inoculated on slants and incubated at 30°C for 72 hr; additionally, adjustments were made to achieve a density of approximately 6.0 × 10^8^ spores/mL. In brief, 5% (*v*/*v*) inocula were transferred into 250 ml Erlenmeyer flasks containing fresh PD (30 ml) media with 50 mg/L of β‐CY. Degradation trials using 100 mg/L 3‐PBA was simultaneously performed. A control experiment was performed by adding the same amount of sterile saline solution followed by incubation for 7 days at 30°C in a rotary shaker at 180 rpm. Concentrations of β‐CY and 3‐PBA residues were determined through high‐performance liquid chromatography (HPLC, LC‐2010C HT, Shimadzu, Kyoto, Japan) according to reported methods (Deng et al., 2015; Liu et al., 2012). The strain that demonstrated the highest degradation efficiency toward β‐CY and 3‐PBA was selected for further studies.

### 2.3 Identification and characterization of strain ET1

A pure isolate, designated as ET1, was selected and identified based on its decomposition efficiency. Colony morphology of strain ET1 on PDA plates was observed and recorded for five consecutive days, and individual morphological characteristics were examined under a light microscope (CX21, Olympus, Tokyo, Japan) following inoculation on a PDA plate and incubation at 30°C for 72 hr.

Total genomic DNA was extracted using DNA extraction kits (Tiandz Biotechnology Co., Ltd, Beijing, China). ITS sequence was amplified through PCR (C1000 Thermal Cycler, Bio‐Rad, Hercules, USA) by using the universal primers ITS1 (5′‐TCCGTAGGTGAACCTGCGG‐3′) and ITS4 (5′‐TCCTCCGCTTATTGATATGC‐3′). PCR products were T‐cloned and sequenced after purification by Biological Engineering Co., Ltd. (Dalian, China). The obtained sequences were compared with the sequences in the GenBank database by BLAST. Phylogenetic tree was analyzed and constructed through neighbor‐joining method by using MEGA version 5.2.

### 2.4 Biodegradation of β‐CY by strain ET1

#### 2.4.1 Relationship between growth characteristics of strain ET1 and β‐CY degradation

Biodegradation process is closely linked to microbial biomass. β‐CY degradation experiments were performed in 250‐ml Erlenmeyer flasks containing 30 ml fresh PD and PD‐β‐CY (concentration of β‐CY was 50 mg/L). Technically, the inoculum level of ET1 in all experiments was 5% (*v*/*v*, 6.0 × 10^8^ spores/mL) unless otherwise stated. After inoculating, cultures were incubated at 30°C in a rotary shaker at 180 rpm for 8 days. Control experiment was prepared with the same amount of sterile saline solution. Each experiment was conducted in triplicate. Samples were collected daily during the period. Growth characteristics of strain ET1 were determined by the dry weight of mycelia, which were obtained through filtering followed by drying at 80°C. Residual β‐CY concentration was measured by HPLC, and the operating conditions were similar to those previously described (Deng et al., 2015; Liu et al., 2012).

#### 2.4.2 Degradation kinetics of β‐CY

Changes in environmental conditions significantly affect microbial growth and metabolism, fate and behavior of chemical toxicants in the natural environment will also be affected (Singh, Walker, & Wright, 2006). Some independent variables were tested to explore the degradation characteristics of β‐CY by strain ET1, including substrate concentration (20, 50, and 100 mg/L), incubation temperature (25°C, 30°C, and 35°C), and pH (5.0, 6.0, and 7.0). For all kinetic experiments, samples were withdrawn at indicated times (0, 1, 2, 3, 4, 5, 6, 7, and 8 days), and triplicate replications were conducted. Residual β‐CY concentrations were determined according to the method described above, and data were fitted to a first‐order kinetics equation (Cycoń, Wójcik, & Piotrowska‐Seget, 2009).

### 2.5 Biodegradation of 3‐PBA

Although experiments on 3‐PBA biodegradation by strain ET1 were roughly analogous to those on β‐CY degradation, some differences were remarkable, especially substrate concentration. It was 100 mg/L in microbial growth experiment, and switched to 50, 100, and 150 mg/L^,^ respectively when it comes to kinetic studies.

### 2.6 Identification of β‐CY and 3‐PBA metabolites

Given the high degradation rate of 3‐PBA and the chemical property of its probable metabolites, researches on degradation pathway were divided into two parts.

#### 2.6.1 GC‐MS analysis

1. Inocula were carefully transferred into 30 ml of fresh PD‐β‐CY medium (50 mg/L) and incubated at 30°C in a rotary shaker at 180 rpm for 8 days. Samples were totally taken from each culture daily, mixed with an equal volume of acetonitrile, and ultrasonically extracted (40 kHz, 300 W) for 30 min. Afterward, 10 ml of solutions obtained from the samples was combined and then centrifuged (10,000 × *g*) for 15 min.
2. Supernatant was concentrated by using a pressured nitrogen‐blowing concentrator under moderate stream, and the residue was dissolved in an appropriate volume of acetonitrile. The resultant solution was centrifuged at 10,000 × *g* for 15 min, and supernatant was dehydrated by anhydrous Na_2_SO_4_.
3. After dehydration, the sample was centrifuged (10,000 × *g*) for 15 min, and supernatant was filtered using a 0.45 μm membrane prior to performing gas chromatography‐mass spectrometry (GC‐MS, 7890A/5975C, Agilent, Santa Clara, USA) analysis.

The GC‐MS was equipped with a HP‐5MS capillary column (30 m × 0.25 mm, 0.25 μm), and operating conditions were based on those used by Chen et al. (2014).

#### 2.6.2 LC‐MS analysis

After transferring inocula into 30 ml of fresh PD‐3‐PBA medium (100 mg/L). The methods used in sampling and pretreatment were similar to those previously described (Deng et al., 2015; Liu et al., 2012).

The sample was subjected to separation by an HPLC system (1,260, Agilent, Santa Clara, USA) equipped with an Agilient Poroshell 120 EC‐C18 analytical column (50 × 4.6 mm, 2.7 μm). The injection volume of samples was 10 µl. Isocratic elution was performed by using acetonitrile and water (60:40, *v*/*v*) as mobile phases at a flow rate of 1.0 ml/min. The HPLC system was connected to a triple quadrupole mass spectrometry (QqQ‐MS, G6460C, Agilient, Santa Clara, USA) equipped with an electrospray interface operating in the positive (ESI^+^) and negative (ESI^−^) modes. The operation parameters were as follows: capillary voltage, positive/4,000 V, negative/−3,800 V; nebulizer pressure, 45 psi; drying gas flow rate, 13 L/min; and gas temperature, 350°C.

Also, those suspected metabolites according to the analyzing results were set as substrate (50 mg/L) for degradation experiments, to reconfirm the biochemical pathway. The analysis conditions were similar to those used by Li et al. (2016).

### 2.7 Data analysis

Degradation rate was calculated by Equation (1):

$$\text{Degradation rate (\textbackslash{}\% ) =}\;\frac{C_{0}-C}{C_{0}}\times 100,>$$

where *C*_0_ is the initial concentration of substrates in samples (mg/L), and *C* represents the residual concentration of substrates in sample solutions (mg/L).

Degradation rate constant (*K*_d_) was determined using the kinetic model $\ln S=-tK_{d}+\ln S_{0}>$, where *S*_0_ is the initial concentration of substrate, *S* represents residual concentration of substrate at time *t*. *K*_d_ and *t *are the rate constant (mg L^−1^ day^−1^) and degradation period in days, respectively. The half‐lives (*t*_1/2_) of substrate were calculated using the algorithm $t_{1/2}=\frac{\ln2}{K_{d}}>$.

Mean and standard deviation of the degradation rate in each trial were calculated and subjected to ANOVA. Statistical significance was determined using SPSS V22.0.

## 3 RESULTS

### 3.1 Isolation and identification of strain ET1

Supporting Information Table S1 shows 20 strains of golden flower fungi isolated from fu brick tea. These strains demonstrated different abilities of degrading 100 mg/L 3‐PBA in PD media, and the degradation rates ranged from 25.16% to 99.99%. Among them, only AT3, ET1, and GT2 were able to degrade β‐CY. Strain ET1 was chosen in the subsequent experiments since it exhibited the highest degradation rate.

Supporting Information Figures S1 and S2 show the colony and morphological characteristics of strain ET1, respectively. As can be seen, the colonies on PDA plates spread broadly after 5 days, which showed an irregularly round shape with a roughened surface, achieving a diameter of 45.0 mm (Supporting Information Figure S1a). The central region of the colonies gradually turned black‐brown while the periphery remained light yellow due to very abundant production cleistothecia, which were spherical or subsphaeroidal (Supporting Information Figure S2a). Besides, the edges of the colonies showed ivory color. When observing from the reverse side, the pigment produced during growth permeated the medium (Supporting Information Figure S1b). Mycelia were apparently septate. Ascus, as well as conidia, was both observed. (Supporting Information Figure S2b,c).

The ITS gene of strain ET1 was amplified, and a 506 bp single fragment (Supporting Information Figure S3) was obtained and then sequenced (GenBank Accession No. KF317836). BLAST analysis indicated that the ET1 sequence was highly similar (99%) to the ITS sequences of GU784865.1 and EF652078.1 strains of the *E. cristatum* group. A phylogenetic tree was subsequently constructed (Supporting Information Figure S4). Strain ET1 was eventually identified as *E*. *cristatum* (Collection number: CGMCC 8293) based on colony, morphological characteristics, and ITS sequence analyses.

### 3.2 Biodegradation of β‐CY

#### 3.2.1 Growth characteristics of strain ET1 and degradation of β‐CY

**Figure 1:**
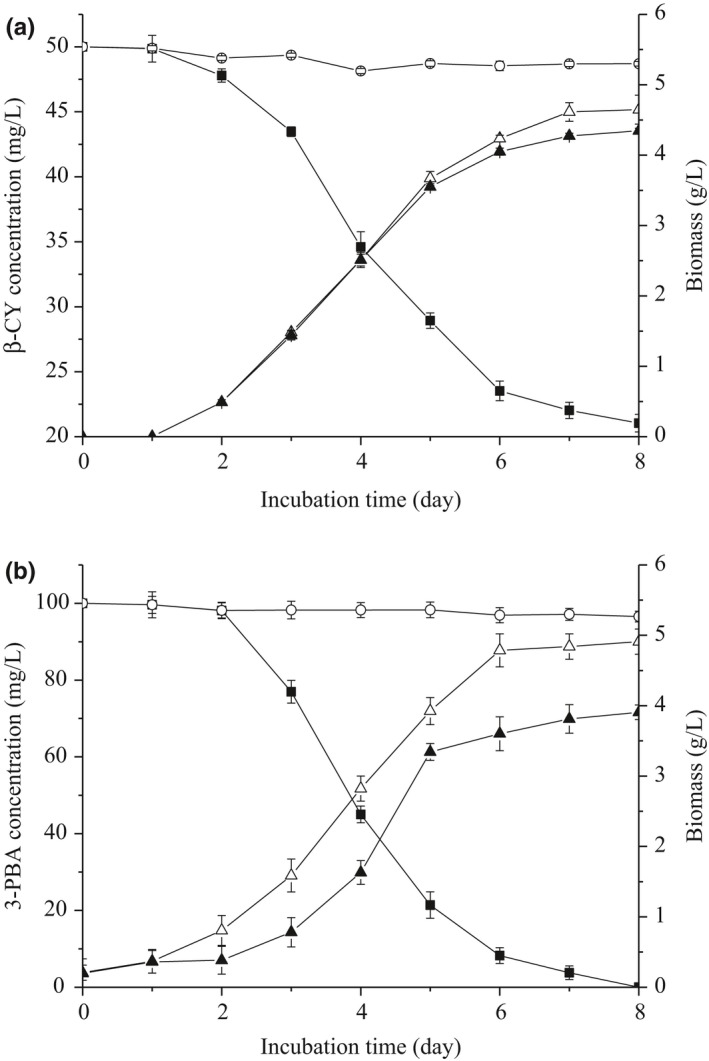
Curves of ET1 biomass and degradation of substrates by strain ET1. Symbols: (▲) biomass in PD‐substrate medium, (△) biomass in PD medium, (■) residual substrate concentration, and (○) control. (a) β‐CY; (b) 3‐PBA. Values are means of three replicates with standard deviation

Figure 1a shows the relationship between biomass and biodegradation course. β‐CY did not significantly exert effect on biomass because of few differences in terms of mycelia dry weight were observed between PD and PD‐β‐CY culture systems during incubation. After 8 days, the final biomass of ET1 in PD medium was 4.65 g/L and that in PD‐β‐CY medium was slightly lower (4.35 g/L). The amount of β‐CY decreased a bit initially, followed by rapid degradation after 2 days. As shown, ET1 strain eventually degraded 57.93% of β‐CY.

#### 3.2.2 Degradation kinetics of β‐CY by strain ET1

Results of dynamics studies on β‐CY degradation are given in Table 1, which indicated that the process of β‐CY degradation by strain ET1 fitted well with a first‐order kinetic equation, turned out that *t*_1/2 _of β‐CY was 3.382–11.517 days under tested conditions. Notably, it increased significantly with increasing substrate concentration from 20 to 100 mg/L, whereas few changes were observed under increasing incubation temperature from 25°C to 35°C or under varying pH ranging from 5.0 to 7.0. These features render the agent of pesticide degradation a competitive advantage in a highly variable environment.

**Table 1:** Kinetic parameters of β‐CY and 3‐PBA degradation by strain ET1 under different variables

Substrate	Factors	Value	Regression equation	*T*_1/2_ (day)	*K*_d_ (mg L^−1^ day^−1^)	*R* ^2^
β‐CY	Substrate concentration (mg/L)	20	ln*S* = −0.205*t* + 3.027	3.382	0.205	0.954
		50	ln*S* = −0.113*t* + 3.919	6.125	0.113	0.922
		100	ln*S* = −0.062*t* + 4.616	11.517	0.062	0.927
	Incubation temperature (°C)	25	ln*S* = −0.114*t* + 3.932	6.104	0.114	0.937
		30	ln*S* = −0.113*t* + 3.919	6.125	0.113	0.922
		35	ln*S* = −0.110*t* + 3.921	6.285	0.110	9.914
	pH	5.0	ln*S* = −0.109*t* + 3.935	6.335	0.109	0.927
		6.0	ln*S* = −0.113 *t* + 3. 19	6.125	0.113	0.922
		7.0	ln*S* = −0.107*t* + 3.926	6.496	0.107	0.943
3‐PBA	Substrate concentration (mg/L)	50	ln*S* = −0.413*t* + 3.934	1.678	0.413	0.926
		100	ln*S* = −0.396*t* + 4.619	1.749	0.396	0.912
		150	ln*S* = −0.341*t* + 5.021	2.032	0.341	0.931
	Incubation temperature (°C)	25	ln*S* = −0.351*t* + 4.619	1.974	0.351	0.949
		30	ln*S* = −0.396*t* + 4.619	1.749	0.396	0.912
		35	ln*S* = −0.372*t* + 4.613	1.863	0.372	0.928
	pH	5.0	ln*S* = −0.221*t* + 4.617	3.136	0.221	0.932
		6.0	ln*S* = −0.396*t* + 4.619	1.749	0.396	0.912
		7.0	ln*S* = −0.217*t* + 4.611	3.194	0.217	0.964

### 3.3 Biodegradation of 3‐PBA

#### 3.3.1 Growth characteristics of strain ET1 and degradation of 3‐PBA

Figure 1b shows the differences of ET1 cultured in PD and PD‐3‐PBA media in terms of biomass. In PD medium, strain ET1 presented lag (0–1 days) and logarithmic phases (1–6 days). Biomass of ET1 stabilized after 6 days, and the final cell dry weight was 4.91 g/L. By contrast, ET1 in PD‐3‐PBA displayed a longer lag period (0–2 days) and then grew rapidly (2–5 days), finally achieving a lower (*p* < 0.05) biomass of 3.91 g/L. Degradation rate of 3‐PBA rapidly increased after 2 days though a slight reduction in substrate was initially observed. ET1 strain completely degraded 100 mg/L of 3‐PBA within 8 days.

#### 3.3.2 Degradation kinetics of 3‐PBA by strain ET1

Degradation dynamic results (Table 1) showed that degradation of 3‐PBA by strain ET1 followed a first‐order kinetics, with *R* ^2^ ranging from 0.912 to 0.964. *t*_1/2_ of 3‐PBA slightly changed with increasing substrate concentration from 50 to 150 mg/L or under an increasing incubation temperature from 25°C to 35°C; moreover, a pH of 6.0 was favorable for 3‐PBA degradation. *t*_1/2 _of 3‐PBA varied from 1.749 to 3.194 days within a certain range of the independent variables in the dynamic experiment; such *T*_1/2 _range was considerably shorter than the normal *T*_1/2_ (180 days; Halden et al., 1999). This observation further confirmed that strain ET1 effectively degraded 3‐PBA.

### 3.4 Metabolites of β‐CY and 3‐PBA by strain ET1

#### 3.4.1 Analysis of β‐CY biodegradation metabolites by using GC‐MS

Metabolites in the PD‐β‐CY culture system were extracted and detected by GC‐MS to determine the β‐CY degradation pathway. Two peaks were observed at *m*/*z* values of 198 and 94. These compounds corresponded to 3‐phenoxybenzaldehyde and phenol based on the National Institute of Standards and Technology (USA) library database (Supporting Information Figure S5).

#### 3.4.2 Analysis of 3‐PBA biodegradation metabolites by LC‐MS

To obtain the maximum sensitivity for identification and detection of the target compounds, we carefully optimized some MS parameters, such as collision energy and fragmentor voltage, for each analyte (data not shown). Two peaks with retention times of 2.660 min (Cpd 1) and 1.493 min (Cpd 2) were observed. On the basis of the precursor molecule and previous studies, we speculated that Cpd 1 with product ions detected at *m*/*z* of 168.8 corresponds to 3‐PBA after a carboxyl group [M‐COOH]^−^ is lost, and Cpd 2 corresponds to catechol, by losing a H_2_O molecule [M‐H_2_O]^−^ (Ravber et al.., 2016; Rousis et al., 2016; Supporting Information Figure S6).

Additionally, strain ET1 degraded all the hypothetic metabolites only in PD media. No apparent difference in terms of substrate concentration was observed involving MM medium (Supporting Information Table S2).

## 4 DISCUSSION

Microbes play important roles in contaminants degradation (Cao, Nagarajan, & Loh, 2009). Remarkably, fungi offer a potential application in bioremediation because of their oxidase systems, some of which demonstrate more efficient degrading effects toward xenobiotic substances than bacteria (Harms, Schlosser, & Wick, 2011). The presence of pesticide residues is a major bottleneck in the international trade of tea commodities. For the first time, we screened a novel strain of golden flower fungus, namely, *E*. *cristatum* ET1, from fu brick tea; this strain could thoroughly degrade both β‐CY and its metabolite 3‐PBA, without accumulating any other toxic intermediates. Our findings agree with the previous theory that the manufacturing process of fermentation is important steps in reducing pesticide residues (Bajwa & Sandhu, 2014; Chen, ShangGuan, Wu, Xu, & Fu, 2012a; Sood, Jaggi, Kumar, Ravindranath, & Shanker, 2004). Actually, *E. cristatum*, hailed as “golden flora,” is the dominant beneficial fungal population throughout the fermentation of fu brick tea, in which contribute to the production of characteristic aroma and flavor (Xu et al., 2011). Particularly, this species received increasing attention because of the antitumor and anticancer activities of its secondary metabolites (Almeida et al., 2010). According to our research, strain ET1 degraded 57.93% of β‐CY (50 mg/L) and completely degraded 100 mg/L of 3‐PBA after incubation at 30°C in PD medium for 8 days, demonstrating a considerably superior degrading capacity over the other reported strains, such as *Ochrobactrum* sp. DG‐S‐01 (Chen, Hu, et al., 2011a), *Streptomyces* sp. HP‐S‐01 (Chen, Geng, et al., 2012b), and *Aspergillus niger* YAT (Deng et al., 2015). Based on these advantages, this particular isolate shows promising potential as bioremediation organism for removal of pesticide residues from fermented food, especially black tea. Moreover, other golden flower fungi strains isolated from fu brick tea exhibited different degrees of degradation toward 3‐PBA. Although not as good as ET1, these golden flower fungi strains enrich the 3‐PBA‐degrading microbe pool. This interesting finding corroborates that observed by Zhu et al. (2016), possibly due to the unique ability of filamentous fungi to degrade 3‐PBA, but further investigations are required to confirm this speculation.

The relationships between growth characteristics of ET1 and degradation of β‐CY and 3‐PBA were explored. The results indicated that substrate degradation rate was positively correlated with biomass. Compared with β‐CY, 3‐PBA significantly affected the growth of ET1 in PD. These results coincide with those of Deng et al. (2015) and it could be attributed to the phenomenon wherein 3‐PBA exerts strong toxic effects on fungi (Stratton & Corke, 1982). Degradation rates of β‐CY and 3‐PBA by ET1 were described using a first‐order kinetic model. With regard to substrate, lag phase possibly extended at higher β‐CY concentrations during formation of necessary degradation enzymes; however, complete inhibition or cell death did not occur. Chen et al. (2014) observed a similar phenomenon in which degradation was inhibited at different initial pesticide concentrations. Moreover, strain ET1 efficiently degraded 50–150 mg/L 3‐PBA. These results suggested that strain ET1 was an ideal organism for bioremediation of pesticides‐contaminated environment.

**Figure 2:**
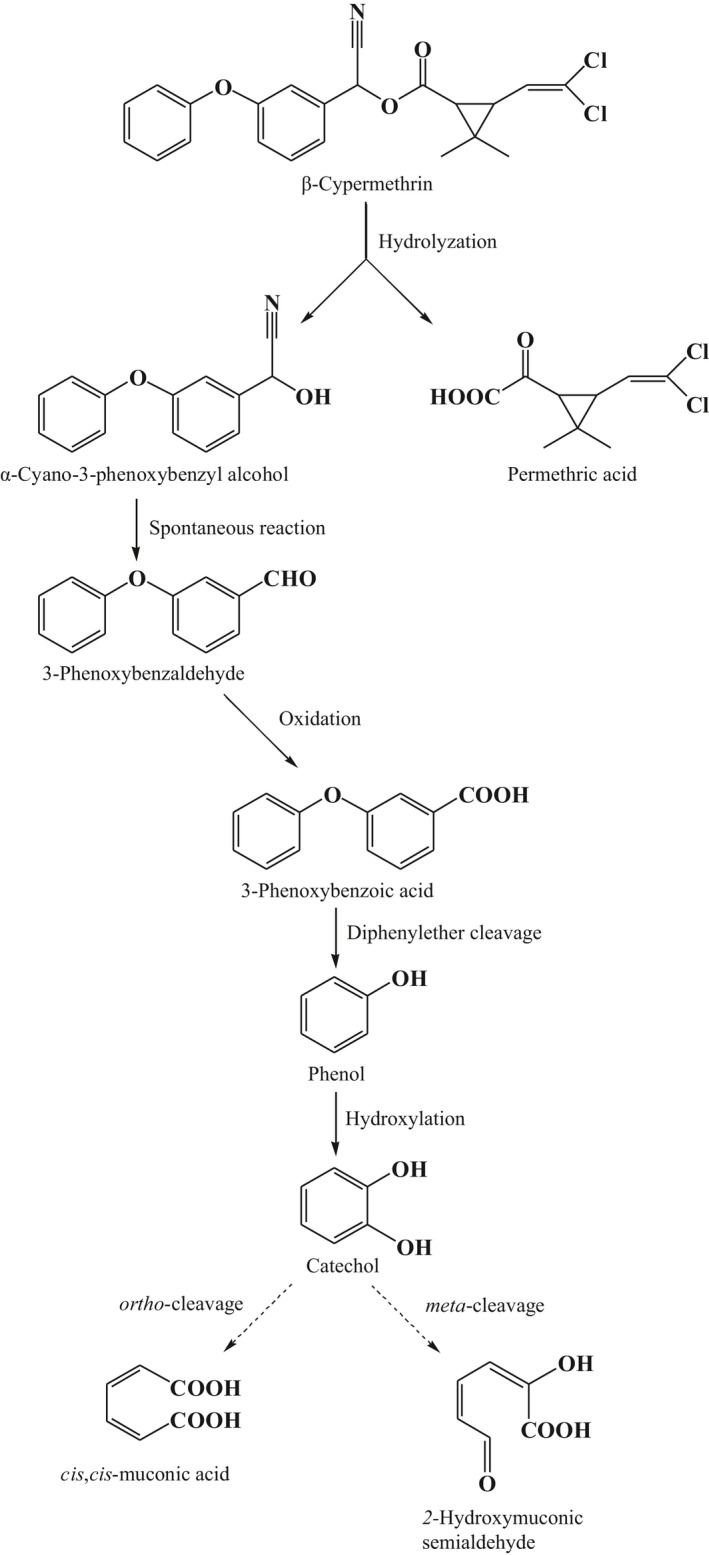
Proposed pathway of β‐CY degradation by strain ET1

During β‐CY and 3‐PBA degradation, three compounds, namely, 3‐phenoxybenzaldehyde, phenol, and catechol, were identified as metabolites based on results of GC‐MS and LC‐MS analyses, which were previously detected (Chen, Hu, et al., 2012c; Deng et al., 2015; Tallur et al., 2008; Zhu et al., 2016). The results evidently showed that strain ET1 transforms β‐CY through hydrolysis to yield α‐cyano‐3‐phenoxybenzyl alcohol, which was very unstable and spontaneously rearranged into 3‐phenoxybenzaldehyde followed by dehydrogenation into 3‐PBA. The initial hydrolysis reaction was similar to that described in other microorganisms and was the most significant step in detoxification of SPs (Deng et al., 2015; Sogorb & Vilanova, 2002; Tallur et al., 2008). Nonetheless, the reported pathways about 3‐PBA biodegradation were obviously different, which could be tentatively categorized into two routes: (a) 3‐PBA is directly degraded into protocatechuic acid and phenol due to the action of dioxygenase; or (b) parent compound is hydroxylated to produce 3‐hydroxy‐5‐phenoxy benzoic acid followed by further metabolism through diphenylether cleavage to yield gallic acid and phenol (Deng et al., 2015; Zhu et al., 2016). Moreover, it has been also observed that 3‐PBA is subject to diaryl ether cleavage, resulting in formation of protocatechuic acid, 3,4‐dimethoxyphenol, and phenol (Chen, Hu, et al., 2012c). About the depletion of model compounds, our results indicated that 3‐phenoxybenzaldehyde, phenol, and catechol were degraded through co‐metabolism (Supporting Information Table S2), and similar phenomena were observed in β‐CY and 3‐PBA (data not shown). Therefore, we concluded that co‐metabolism existed in the entire process of β‐CY degradation by ET1, and this result did not completely agree with previous theory stating that both mineralization and co‐metabolism exist in biochemical degradation of xenobiotics (Deng et al., 2015; Zhu et al., 2016). The reason causing this discrepancy requires further investigation. On the basis of our results and previous findings, we speculated that strain ET1 transformed 3‐PBA into phenol under the action of dioxygenase; phenol was then hydroxylated into catechol, which was further oxidized through the *ortho*‐cleavage or *meta*‐cleavage pathway (Fuchs et al., 2011; Figure 2). However, there is also a possibility that other reactions such as ring‐cleavage occurred before diphenylether cleavage (Kim et al., 2007). Hence, further experiments and additional data are needed to illustrate the complete degradation pathway of β‐CY by ET1, during which the toxicity of metabolites should be evaluated, ascertaining the feasibility in fermented food application.

In summary, a novel *E*. *cristatum* strain ET1 obtained from fu brick tea demonstrated a thorough degradation toward β‐CY and its metabolite 3‐PBA, in which no toxic metabolites accumulated, suggesting that strain ET1 was useful and secure in bioremediation of pesticide‐contaminated environments and fermented food, especially black tea. Moreover, we proposed a microbial metabolic pathway for β‐CY and 3‐PBA, in which hydrolyzation and oxidation play important roles.

## CONFLICT OF INTEREST

We declare that no conflict of interest exists in the submission of this manuscript.

## AUTHORS CONTRIBUTION

Kaidi Hu, Weiqin Deng, and Jinyong Li carried out the experiments, the main conceptual ideas and proof outline of which is devised by Shuliang Liu, who also contributed to data interpretation. Kaidi Hu took the lead in writing the manuscript, with input from Yuanting Zhu and Aiping Liu. All authors provided critical feedback and helped shape the research, analysis, and manuscript.

## ETHICS STATEMENT

None required.

## DATA ACCESSIBILITY

All data are provided in full in this article and Supporting Information, and archived in the Figshare data repository (https://figshare.com/s/431c3603f46fde9b02a0).
