# Supplementary material for: Simultaneous degradation of β‐cypermethrin and 3‐phenoxybenzoic acid by Eurotium cristatum ET1, a novel “golden flower fungus” strain isolated from Fu Brick Tea
Source: Microbiologyopen. 2018 Dec 12;8(7):e00776. doi: 10.1002/mbo3.776 (PMC6612557; doi:10.1002/mbo3.776)
Supplement: Supplementary file 1 [file MBO3-8-e00776-s001.pdf]

Supporting Information

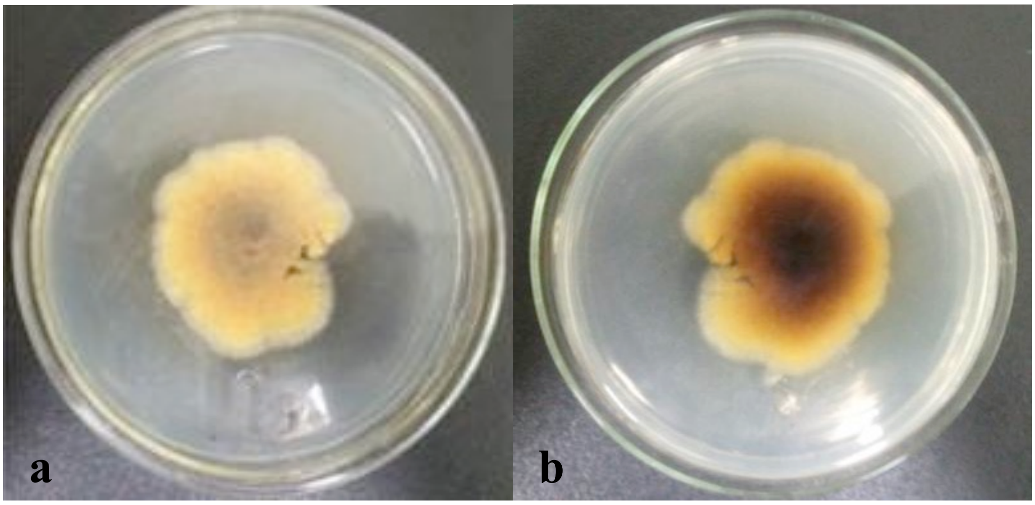

Figure S1 Colony characteristics of strain ET1. a: front; b: back.

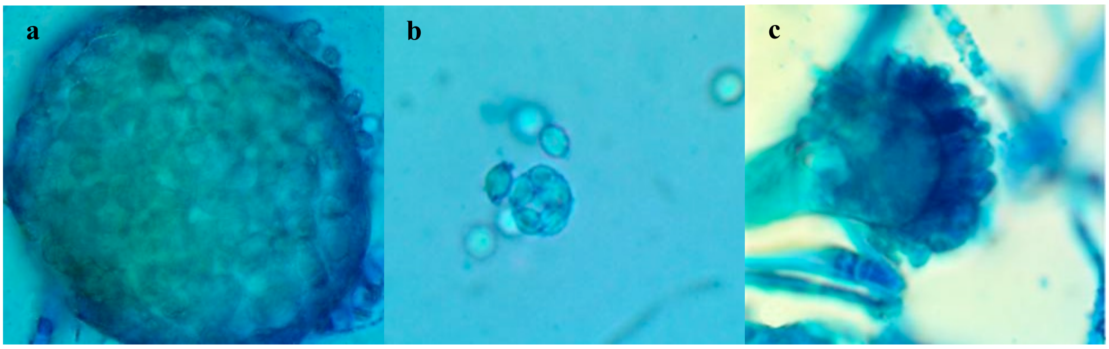

Figure S2 Morphological characteristics of strain ET1. a: cleistothecia; b: ascus; c: mycelia and conidia.

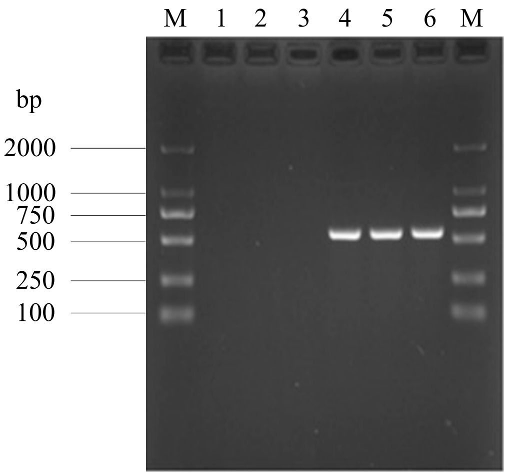

Figure S3 Electrophoretogram of an ITS PCR product of strain ET1. M: DNA marker; 1–2: addition of primer without template; 3: addition of template without primers; 4–6: PCR products.

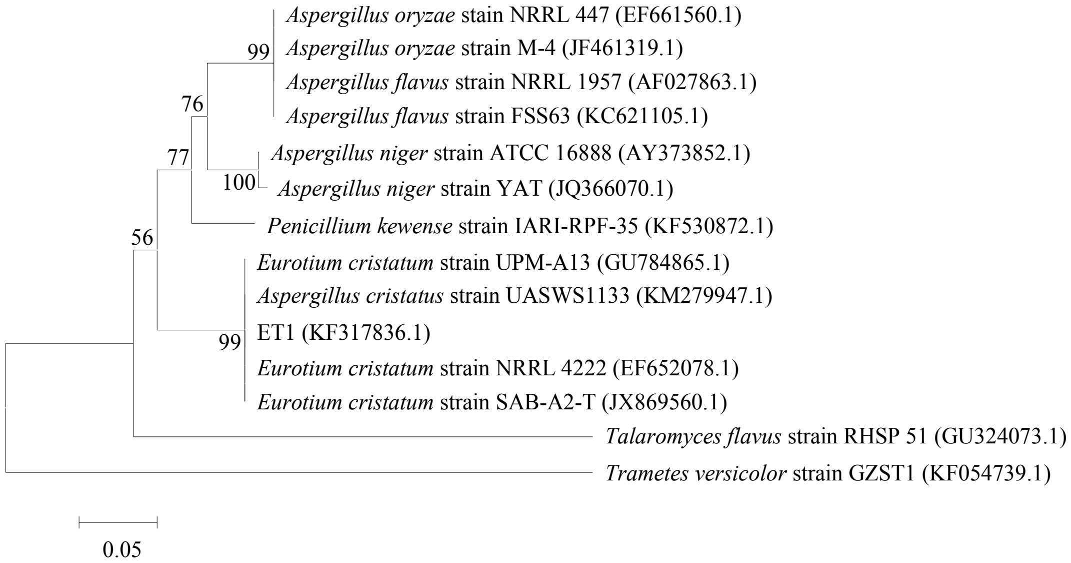

Figure S4 Phylogenetic tree of ET1 constructed based on ITS sequences. Bootstrap values and GenBank accession numbers are provided.

a

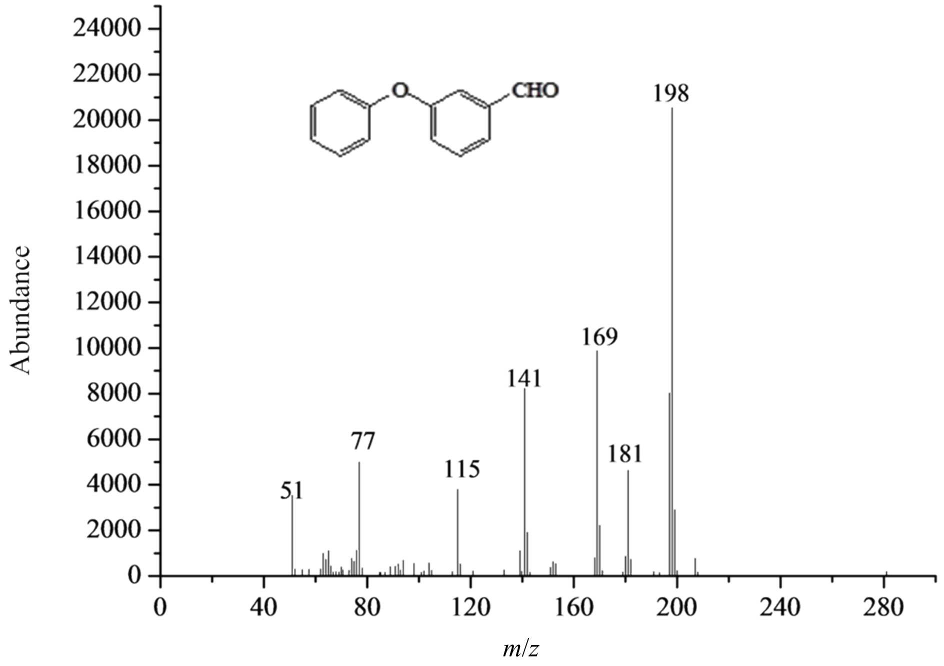

b

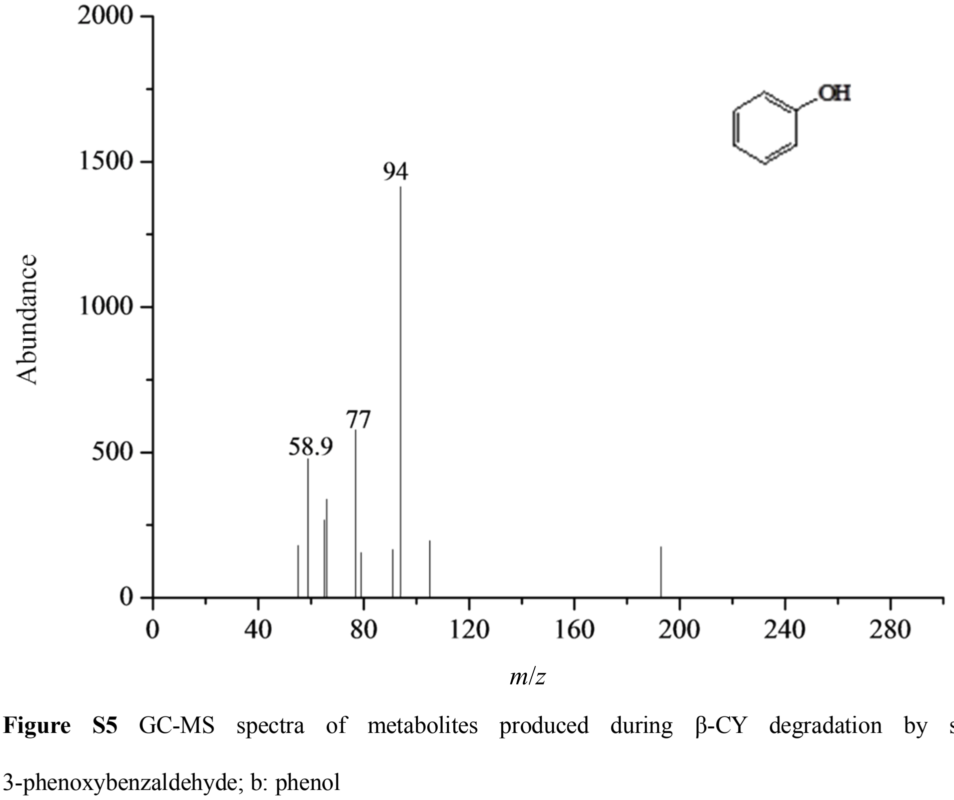

Figure S5 GC-MS spectra of metabolites produced during β-CY degradation by strain ET1. a: 3-phenoxybenzaldehyde; b: phenol

a

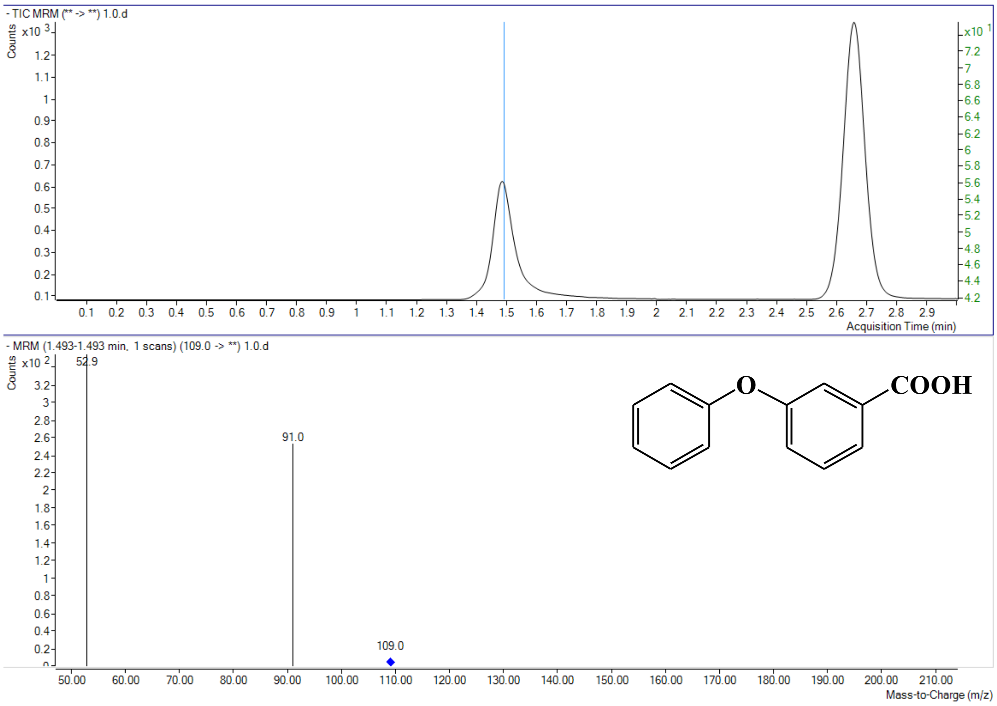

b

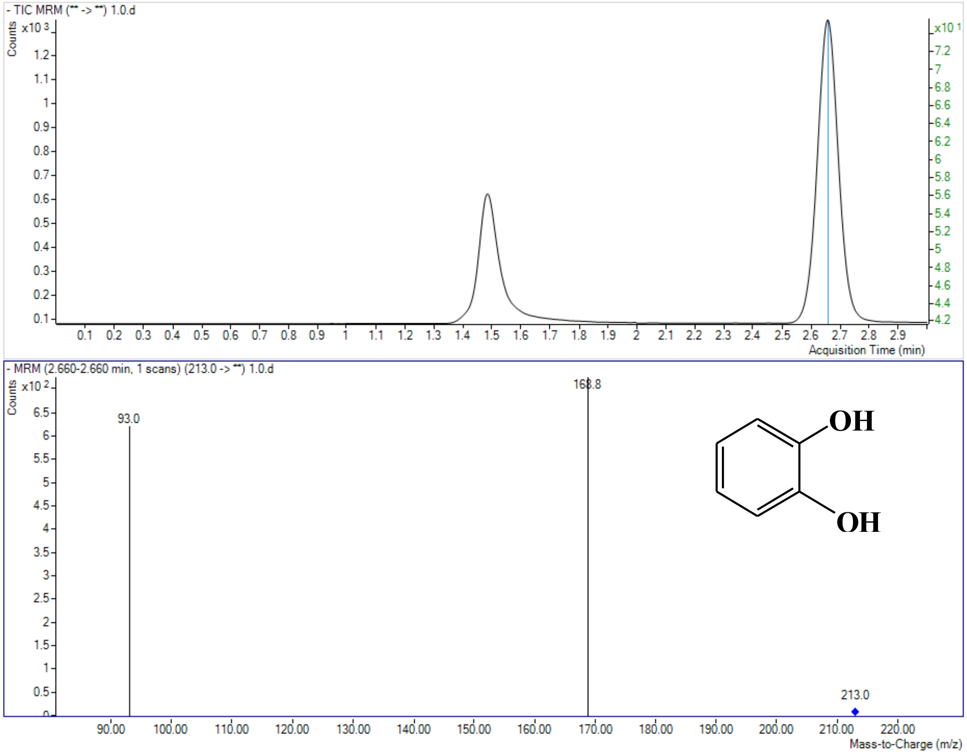

Figure S6 MS/MS spectrums of metabolites produced during 3-PBA degradation by strain ET1. a: 3-PBA; b: catechol. *m/z* (s) of precursor molecule and product ions are provided.

Table S1 Degradation of β-CY and 3-PBA by isolated strains

Number	Source of fu brick tea	Degradation rate of β-CY (%)	Degradation rate of 3-PBA (%)
AT1	Anhua Tea Factory	—	32.35
AT2		—	41.54
AT3		23.2	67.39
AT4		—	56.62
ET1	Yiyang Tea Factory	54.1	99.99
ET2		—	75.23
ET3		—	39.56
ET4		—	67.32
ET5		—	39.54
ET6		—	59.57
ET7		—	79.54
BT1	Baishaxi Tea Factory	—	63.15
BT2		—	38.47
BT3		—	49.65
BT4		—	74.23
BT5		—	25.16
GT1	Guanlongyu Tea Factory	—	36.49
GT2		25.7	85.35
GT3		—	63.18
GT4		—	27.54

Note: "—" means no degradation ability was observed

Table S2 Utilization of model compounds by strain ET1

Substrates	MM		PD	
	Biomass (dry cell weight g L^{-1})	Degradation rate (%)	Biomass (dry cell weight g L^{-1})	Degradation rate (%)
3-phenoxybenzaldehyde	0.04	—	3.924	79.47
catechol	0.02	—	4.312	46.33
phenol	0.05	—	3.812	41.29

Note: "—" means no degradation occurred
