# Supplementary material for: Simultaneous degradation of β‐cypermethrin and 3‐phenoxybenzoic acid by Eurotium cristatum ET1, a novel “golden flower fungus” strain isolated from Fu Brick Tea
Source: Microbiologyopen. 2018 Dec 12;8(7):e00776. doi: 10.1002/mbo3.776 (PMC6612557; doi:10.1002/mbo3.776)
Supplement: Supplementary file 2 [file MBO3-8-e00776-s002.docx]

**Figure S1** Colony characteristics of strain ET1. a: front; b: back.

**Figure S2** Morphological characteristics of strain ET1. a: cleistothecia; b: ascus; c: mycelia and conidia.

**Figure S3** Electrophoretogram of an ITS PCR product of strain ET1. M: DNA marker; 1–2: addition of primer without template; 3: addition of template without primers; 4–6: PCR products.

**Figure S4** Phylogenetic tree of ET1 constructed based on ITS sequences by using the neighbor-joining method analysis of 1, 000 resampled data sets. Bootstrap values and GenBank accession numbers are provided. Bar represents sequence divergence.

**Figure S5** GC-MS spectra of metabolites produced during β-CY degradation by strain ET1. a: 3-phenoxybenzaldehyde; b: phenol

**Figure S6** MS/MS spectrums of metabolites produced during 3-PBA degradation by strain ET1. a: 3-PBA; b: catechol. m/z(s) of precursor molecule and product ions are provided.
